# Supplementary material for: L‐OPA1 regulates mitoflash biogenesis independently from membrane fusion
Source: EMBO Rep. 2017 Feb 7;18(3):451–63. doi: 10.15252/embr.201642931 (PMC5331265; doi:10.15252/embr.201642931)
Supplement: Supplementary file 1 — Expanded View Figures PDF [file EMBR-18-451-s001.pdf]

Expanded View Figures

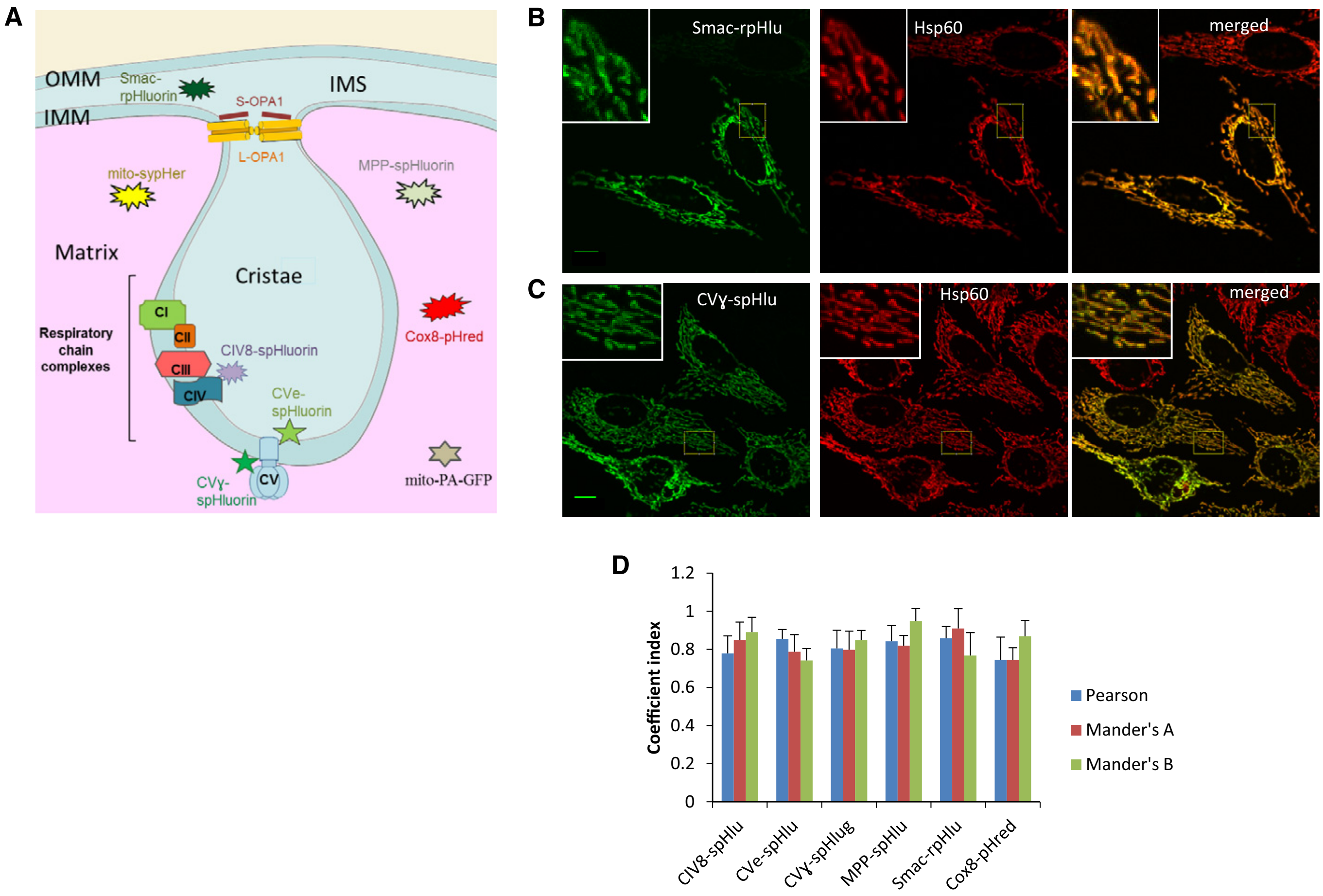

Figure EV1. Mitochondrial localization of the different pH sensors used in this study.

A Predicted mitochondrial localization of pH sensors used in this study. pH-sensitive fluorescent proteins were addressed to the mitochondrial matrix, the intermembrane space (IMS), or the cristae. MPP-spHluorin, mito-sypHer, and Cox8-pHred are expressed in the matrix. Smac-rpHluorin is targeted to the IMS. CIV8-spHluorin is fused to the complex IV subunit 8a and faces the intra-cristae space, while CVe- and CVγ-spHluorins are fused to the complex V and are located on the cristae side and on the matrix side, respectively. Complexes formed by long (L-OPA1) and short forms (S-OPA1) of OPA1 are located at the cristae junctions. OMM: outer mitochondrial membrane, IMM: inner mitochondrial membrane, CI: complex I, CII: complex II, CIII: complex III, CIV: complex IV, CV: complex V.

B–D Colocalization between the endogenous mitochondrial marker Hsp60 and the different pH sensors. Images of HeLa cells showing endogenous Hsp60 (red signal) and (B) Smac-rpHluorin or (C) CVγ-spHluorin (green signal) and their overlays. The insets show higher magnification of the regions outlined by yellow rectangles. Scale bars: 10 μm. (D) Analysis of colocalization is represented by Pearson's coefficient (indicating the correlation between Hsp60 and the pH probe signals) and by the Mander's A (representing the proportion of Hsp60 signal overlapping with the pH probes) and Mander's B coefficients (representing the proportion of the pH probe signals overlapping with Hsp60). Data are means ± SD of three independent experiments.

 © 2017 The Authors

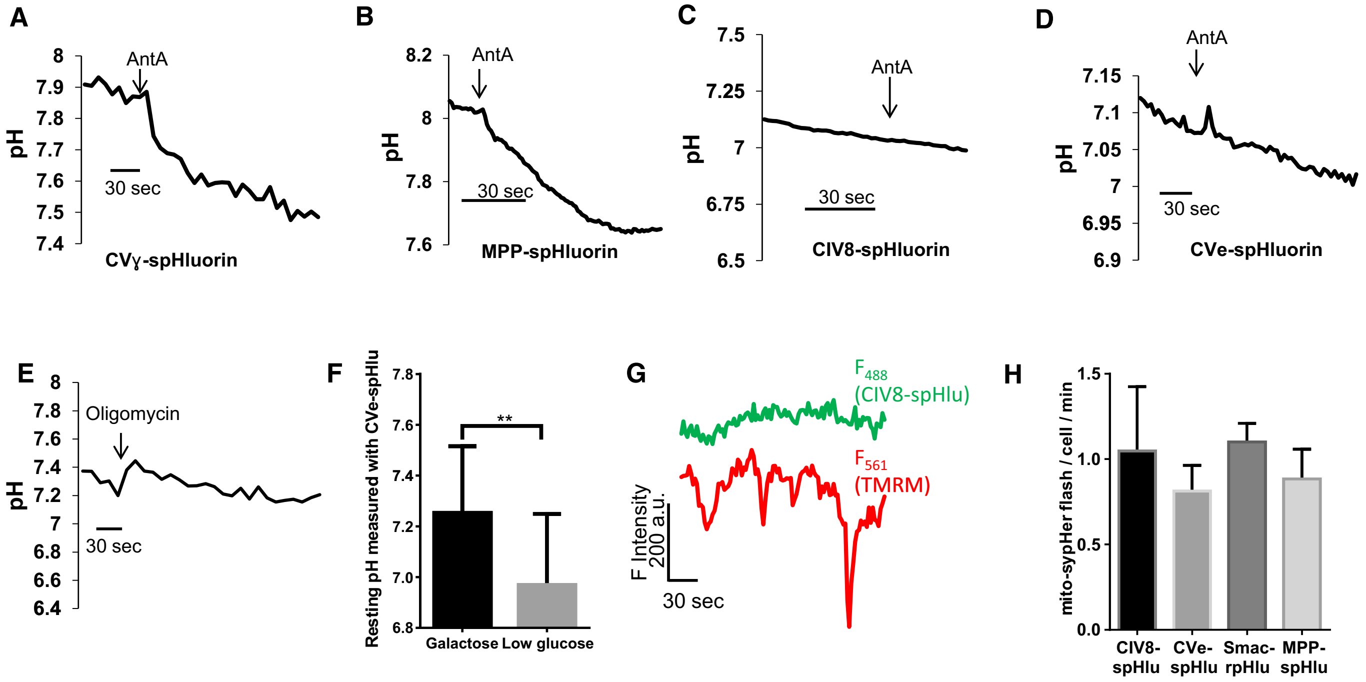

Figure EV2. The IMS and cristae probes report chronic, but not acute pH changes.

A–D Effect of antimycin in HeLa cells expressing spHluorins targeted to matrix or cristae. Change in fluorescence intensity of (A) CVγ-, (B) MPP-, (C) CIV8-, and (D) CVe-spHluorins (λ_{ex}: 488 nm) evoked by the addition of antimycin A (AntA). Fluorescence decreases correspond to an acidification of the mitochondrial compartment.

E Effect of oligomycin in HeLa cells expressing Smac-rpHluorin.

F Effect of galactose and low glucose media on resting pH values measured with CVe-spHluorin (n = 20 and 19 cells for each medium, respectively). Values are mean ± SD of three independent experiments. Unpaired t-test with Welch's correction, $^{**}P$ = 0.0023.

G CIV8-spHluorin recordings showing the absence of pH transients during drops in $\Delta\psi_m$ in cells cultured with galactose. Identical results were obtained with CVe-spHluorin and Smac-rpHluorin.

H MitopHlash frequency in HeLa cells co-expressing mito-sypHer and Smac-rpHluorin or spHluorins fused to CIV8, CVe, or MPP. mito-sypHer flashing activity persisted unabatedly in cells expressing pH sensors in the intra-cristae space or in the IMS. $^{**}P < 0.01$.

© 2017 The Authors

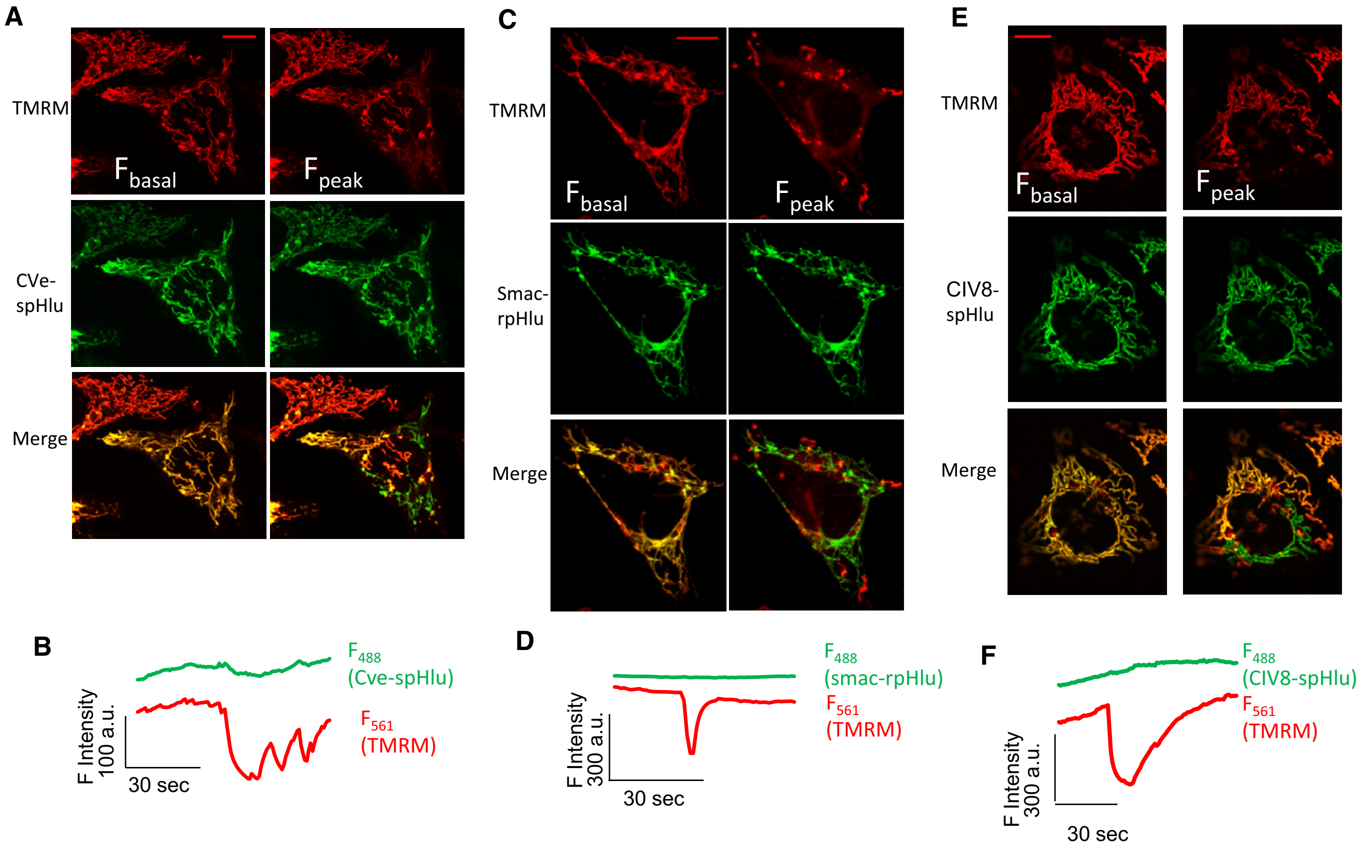

Figure EV3. Simultaneous $\Delta\psi_m$ and pH recordings in HeLa cells expressing DRP1^{K38A}.

A–F Confocal images of cells expressing (A) CVe-spHluorin, (C) Smac-rpHluorin, or (E) CIV8-spHluorin (green signals) loaded with TMRM (red), and time-resolved recordings of changes in TMRM and (B) CVe-spHluorin, (D) Smac-rpHluorin, or (F) CIV8-spHluorin fluorescence. The mitochondrial area of depolarization is greatly enhanced in cells expressing DRP1^{K38A}, but pH changes remain undetectable with the pHluorins. Scale bars: 10 µm.

 © 2017 The Authors

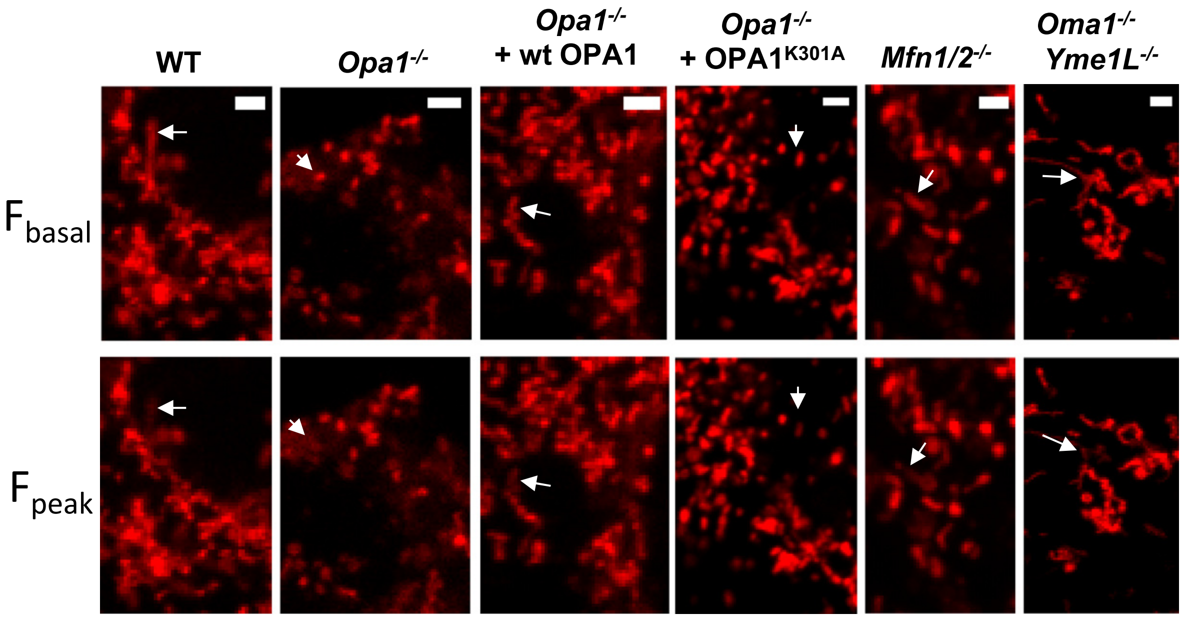

Figure EV4. $\Delta\psi_m$ recordings in MEF cells.
Depolarizations were recorded in WT MEFs, *Opa1*$^{-/-}$, *Opa1*$^{-/-}$ re-expressing WT OPA1 or OPA1^{K301A}, *Mfn1/2*$^{-/-}$ and *Oma1*$^{-/-}$ *Yme1L*$^{-/-}$ cells loaded with TMRM. The arrows indicate the depolarized mitochondria. Scale bars: 2 µm.

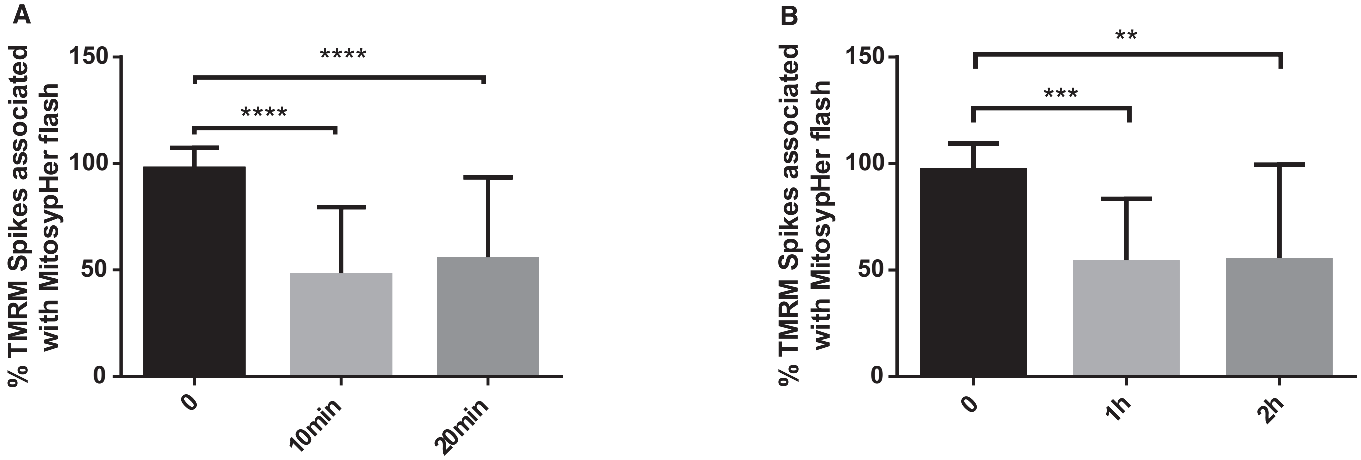

Figure EV5. mitopHlash/$\Delta\psi_m$ uncoupling in cells exposed to H_2O_2 and etoposide.

A, B mitopHlash/$\Delta\psi_m$ coupling in WT MEF cells loaded with TMRM and exposed to (A) 250 mM H_2O_2 for 10 and 20 min or (B) 100 µM etoposide for 1 or 2 h. n = 38, 97, 98 depolarization events recorded in 25, 18, and 22 cells at 0, 10, and 20 min of H_2O_2 treatment, respectively, and n = 27, 111, 34 depolarization events recorded in 16, 18, and 13 cells at 0, 1, and 2 h of etoposide treatment, respectively. Values are means ± SD of three independent experiments. One-way ANOVA with multiple comparisons, $^{**}P$ = 0.0018, $^{***}P$ = 0.0005, $^{****}P < 0.0001$.

© 2017 The Authors
