# Supplementary material for: L‐OPA1 regulates mitoflash biogenesis independently from membrane fusion
Source: EMBO Rep. 2017 Feb 7;18(3):451–63. doi: 10.15252/embr.201642931 (PMC5331265; doi:10.15252/embr.201642931)
Supplement: Supplementary file 2 — Movie EV1 [file EMBR-18-451-s002.zip › EMBOR201642931V3_movie_EV1/EMBOR-2016-42931V3_movie_EV1_legend.docx]

**Movie EV1: mitopHlash recordings in HeLa cells expressing the ratiometric sensor Cox8-pHred.** Time-lapse sequence of F_561_/F_405_ fluorescence ratio in HeLa cells expressing Cox8-pHred. Spontaneous alkalinization transients appear in red and occur repeatedly in individual mitochondria. Blue colour denotes high ratio values and red low ratio values.
