# Supplementary material for: L‐OPA1 regulates mitoflash biogenesis independently from membrane fusion
Source: EMBO Rep. 2017 Feb 7;18(3):451–63. doi: 10.15252/embr.201642931 (PMC5331265; doi:10.15252/embr.201642931)
Supplement: Supplementary file 3 — Movie EV2 [file EMBR-18-451-s003.zip › EMBOR201642931V3_movie_EV2/EMBOR-2016-42931V3_movie_EV2_legend.docx]

**Movie EV2. Simultaneous Δψ_m_ and pH recordings in HeLa cells expressing MPP-spHluorin.** Time-lapse sequence of TMRM (red, left, λex: 561 nm) and MPP-spHluorin (right, λex: 488 nm) fluorescence in HeLa cells. Matrix alkalinization transients, corresponding to increases in pHluorin yellow signal, coincide with drops in Δψ_m_ (decrease of TMRM fluorescence) in different mitochondria. Blue and yellow correspond to low and high fluorescence intensity.
