# Supplementary material for: L‐OPA1 regulates mitoflash biogenesis independently from membrane fusion
Source: EMBO Rep. 2017 Feb 7;18(3):451–63. doi: 10.15252/embr.201642931 (PMC5331265; doi:10.15252/embr.201642931)
Supplement: Supplementary file 4 — Movie EV3 [file EMBR-18-451-s004.zip › EMBOR201642931V3_movie_EV3/EMBOR-2016-42931V3_Movie_EV3_legend.docx]

**Movie EV3.** **Simultaneous Δψ_m_ and pH recordings in HeLa cells expressing CIV8-spHluorin.** Time-lapse sequence of TMRM (red, left, λex: 561 nm) and CIV8-spHluorin (green, right, λex: 488 nm) fluorescence in HeLa cells. No change in pH, corresponding to the green signal was detected during drops in Δψ_m_ (decrease of TMRM fluorescence). Bar: 10 µm.
